# Metastatic Mammary-Like Adenocarcinoma of the Vulva Responding to Cyclin-Dependent Kinase (CDK) Inhibitor: A Case Report and Literature Review

**DOI:** 10.7759/cureus.93598

**Published:** 2025-09-30

**Authors:** Filipa Pereira, Joana L Pimenta, Mariana Rei, Carla R Dias, Deolinda Pereira

**Affiliations:** 1 Medical Oncology, Instituto Português de Oncologia do Porto Francisco Gentil, Entidade Pública Empresarial (EPE), Porto, PRT; 2 Gynecology, Instituto Português de Oncologia do Porto Francisco Gentil, Entidade Pública Empresarial (EPE), Porto, PRT; 3 Pathology, Instituto Português de Oncologia do Porto Francisco Gentil, Entidade Pública Empresarial (EPE), Porto, PRT

**Keywords:** case report, cdk inhibitor, mammary-like gland, rare vulvar adenocarcinoma, vulvar adenocarcinoma

## Abstract

Mammary-like adenocarcinoma originating in the vulva is extremely rare, even rarer when metastasized at diagnosis. It may arise from the primitive mammary ridge or mammary-like glands of the skin in the anogenital region. We report a case of a 47-year-old woman with diffuse bone metastasis and a vulvar lesion compatible with mammary-type adenocarcinoma, with no evidence of primary breast cancer. Systemic treatment followed protocols for hormone receptor (HR)-positive, human epidermal growth factor receptor 2 (HER2)-negative advanced breast cancer, using an aromatase inhibitor and ribociclib, resulting in a favorable clinical and metabolic response. Documenting this case contributes to the understanding and clinical management of this rare neoplasm.

## Introduction

The carcinoma of the vulva accounts for 4% of gynecological neoplasms, and the most common histological type is squamous cell carcinoma. Vulvar adenocarcinomas occur in 0.1% of cases and, within this group, are included mammary gland-like adenocarcinomas [1].

The primitive mammary ridge, which derives from the ectoderm, appears during the fifth week of embryonic development and extends from the axillary region to the inguinal region bilaterally, including the vulva. It regresses, except for the pectoral region, but around 6% of the population maintains ectopic breast tissue along this line [2,3]. This tissue is responsive to hormonal stimuli, just like breast tissue, so there are cases of adenocarcinoma originating in these regions, and when it happens in the vulva, it originates as a mammary-like adenocarcinoma. There is also an alternative theory, which links the origin of vulvar mammary-like glands to variants of skin glands in the anogenital region [4].

Mammary-like adenocarcinomas of the vulva are extremely rare, with around 45 cases reported to date [1,2,5-7]. Given the extreme rarity of this tumor, particularly in a metastatic presentation, there are no standardized treatment guidelines. This report describes a case of metastatic mammary-like adenocarcinoma and details its successful management with a cyclin-dependent kinase (CDK) 4/6 inhibitor-based regimen, providing a valuable therapeutic precedent for the oncology community.

## Case presentation

The patient is a 47-year-old woman, multiparous, pre-menopausal, with a history of stage pT1b melanoma of the left arm, who underwent surgery and sentinel node biopsy in 2010, and has since been under surveillance with no signs of recurrence. She had no other significant personal or family history.

**Figure 1:**
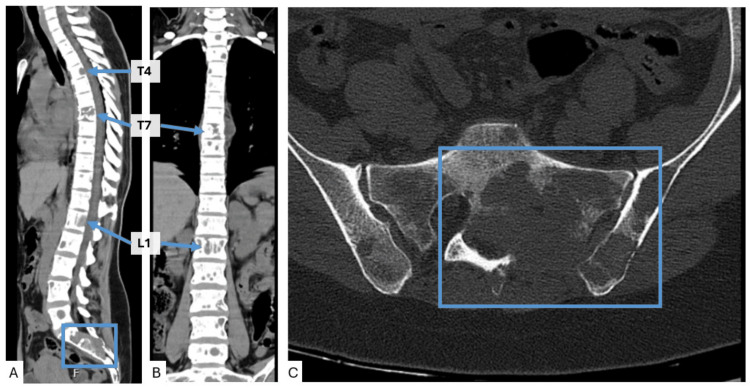
Computed tomography scan of the spine in June 2024, showing scattered lytic lesions throughout the vertebrae. (A) Sagittal plane of the spine; (B) coronal plane of the spine; (C) axial plane of the spine. The images show focal areas of lytic lesions in all vertebral segments, consistent with diffuse bone metastasis seen in images A and B. We can see erosion of the posterior vertebral wall of the T4 vertebral body due to a lytic lesion with tissue component, without invasion of the spinal canal; pathological compression fracture of the T7 vertebral body; erosion of the posterior vertebral wall of L1 due to a lytic lesion with tissue component, without invasion of the spinal canal (all these changes marked with arrows pointing to the specific vertebrae level). There are also extensive osteolytic areas in the sacral region, with a marked associated soft tissue component, easily seen in images A and C (marked with a square).

In June 2024, she began suffering from lumbosacral pain, which prompted a computed tomography (CT) scan of the spine that showed diffuse lytic bone lesions, including a compression fracture at T7 and soft tissue components at T4 and L1 (Figure 1). While these imaging changes were being studied, the patient was admitted due to uncontrolled pain, despite the use of non-steroidal anti-inflammatory drugs (NSAIDs), acute kidney injury, and severe hypercalcemia (grade 4 of Common Terminology Criteria for Adverse Events, version 5 (CTCAE v.5)) with incoercible vomiting. The hypercalcemia was linked to diffuse bone metastasis, and the acute kidney injury to dehydration due to vomiting and NSAID toxicity. The hypercalcemia and renal function resolved after therapy with calcitonin, denosumab, and vigorous fluid therapy. She underwent radiotherapy directed to L1, sacrum, and T4, which, together with optimization of analgesia with opioid therapy, led to resolution of the pain.

**Figure 2:**
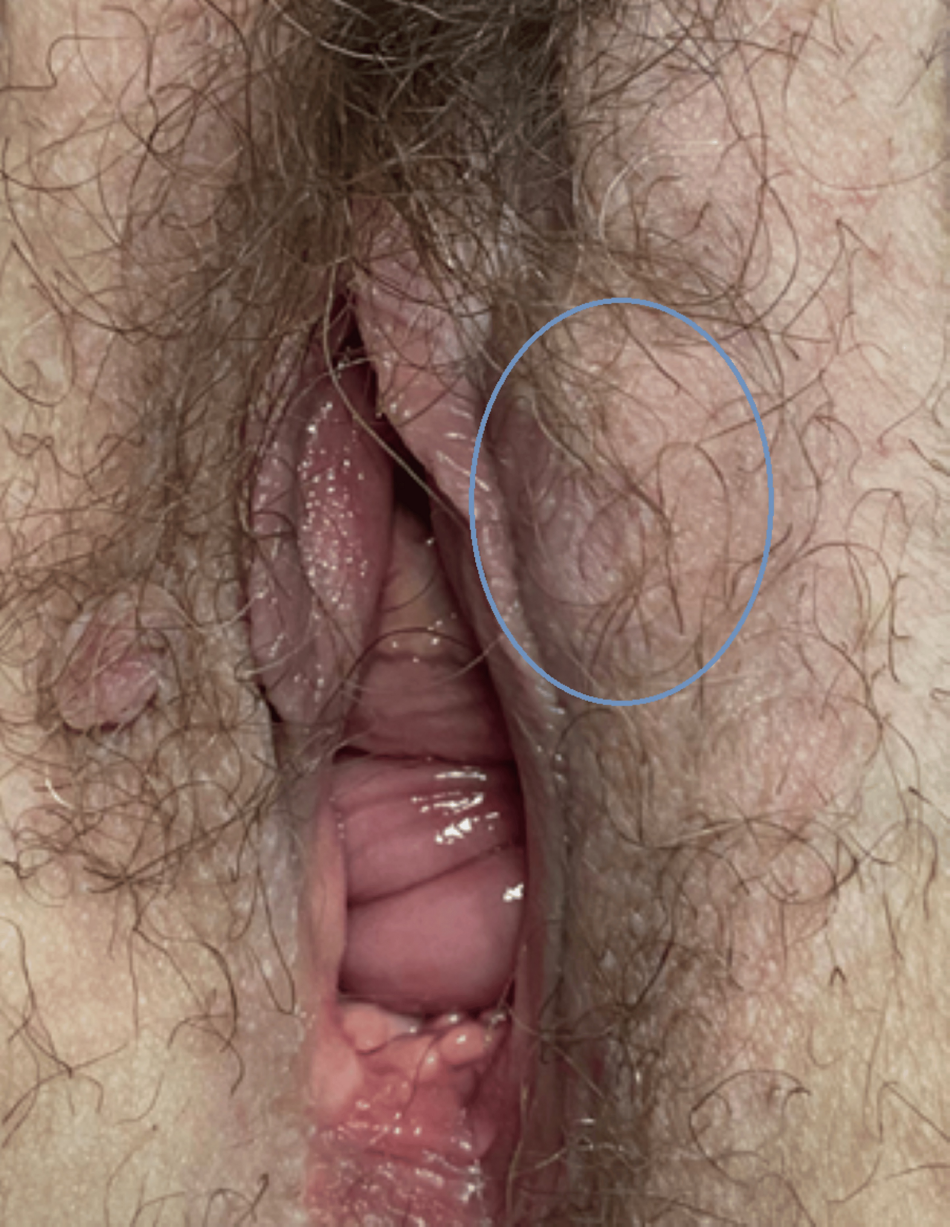
Vulvar swelling on the left labium majus, located deep in the subcutaneous tissue with no cutaneous involvement (outlined by a circle).

During hospitalization, a biopsy of the bone lesion at the iliac level was conducted. The patient also had a hard, irregular vulvar swelling on the left, measuring around 4x2 cm, which she had known about for around a year, with slow growth and associated pain more recently, and it was also assessed and biopsied (Figure 2). A positron emission tomography (PET) scan was carried out, which confirmed the hypothesis of diffuse bone metastasis.

**Figure 3:**
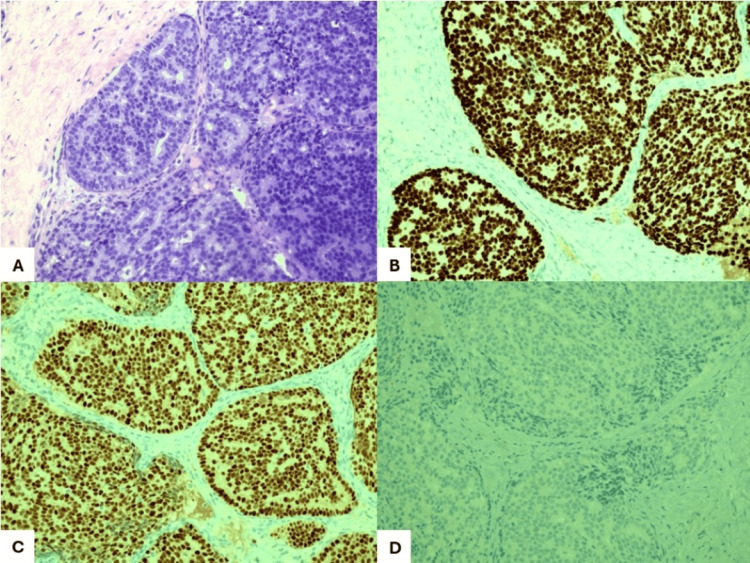
Histologic evaluation of the vulvar biopsy. (A) Neoplasia with cribriform architecture, consisting of cells with rounded and uniform nuclei. (B) The neoplasia shows diffuse immunoreactivity for GATA-binding protein 3 (GATA3). (C) Estrogen receptor (ER) positive in 90-100% of neoplastic cells. (D) The immunohistochemical study for p63 was negative, confirming the absence of myoepithelial cells in the neoplasia and that it is an invasive neoplasia.

Histological examination of the vulvar lesion biopsy was compatible with mammary gland-like adenocarcinoma of the vulva (Figure 3). The immunohistochemical study showed positivity for GATA-binding protein 3 (GATA3), cytokeratin 7 (CK7), estrogen receptors (90% to 100%), progesterone receptors (20% to 30%), and synaptophysin. There was negativity for human epidermal growth factor receptor 2 (HER2), paired box gene 8 (PAX8), tumor protein 63 (p63), and SRY-box transcription factor 10 (SOX10). The bone biopsy was consistent with metastasis of the previously described vulvar adenocarcinoma. This histological type is compatible with a primary carcinoma of the vulva; however, the possibility of metastatic involvement by breast primary should be clinically excluded. The patient underwent breast magnetic resonance imaging (MRI), which ruled out breast disease. She also had a cancer antigen 15-3 (CA 15-3) of 332 U/mL (normal values <25 U/mL).

The case was discussed in a multidisciplinary group, and it was decided to start systemic treatment with an aromatase inhibitor and a CDK 4/6 inhibitor, as indicated in advanced hormone receptor-positive and HER2-negative breast carcinomas, as first-line systemic treatment. Due to the presence of metastatic disease, there was no indication for surgery. The patient started treatment with letrozole 2.5 mg/day, goserelin 3.6 mg every four weeks, and ribociclib 600 mg (28-day cycles, with 21 days of administration of 600 mg/day, followed by a seven-day break) in August 2024. She started concomitant treatment with zoledronic acid due to diffuse bone metastases.

**Figure 4:**
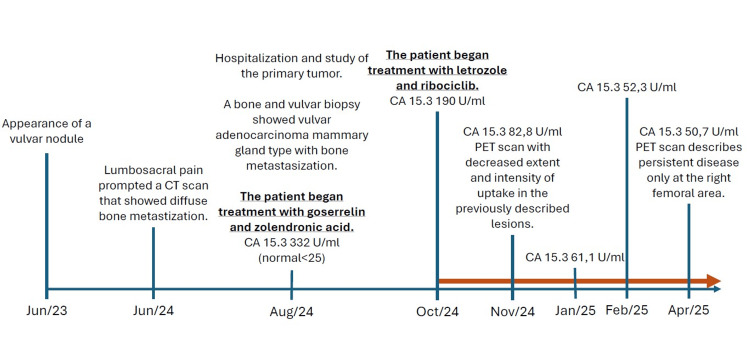
Summary of clinical, imaging, and tumor marker evolution. CA 15-3: cancer antigen 15-3; PET: positron emission tomography

**Figure 5:**
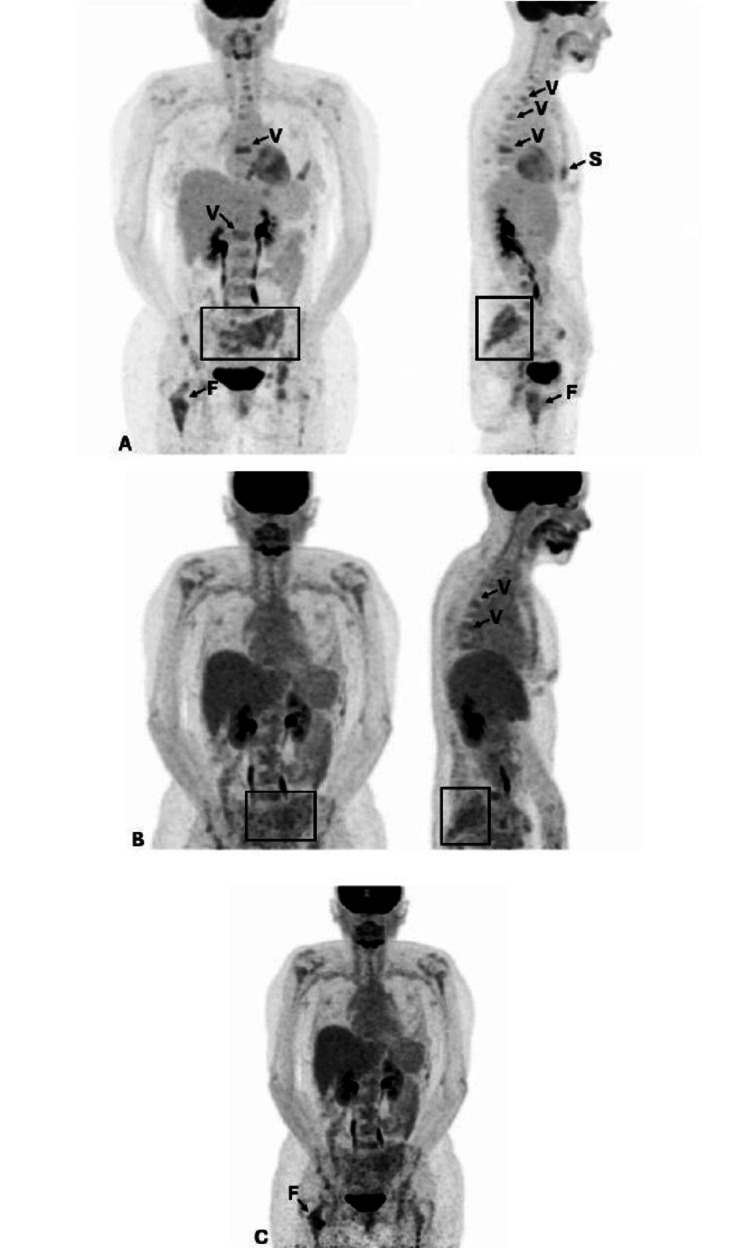
Positron emission tomography (PET) scans done for tumor response evaluation. (A) Baseline PET scan done in June 2024, which showed diffuse bone metastases (vertebral (V), sternal (S), and femoral (F) metastasis) and a sacral soft tissue mass (inside the square). (B) PET scan done in November 2024, after beginning systemic treatment, which showed decreased extent and intensity of uptake in the previously described lesions. (C) PET scan done in April 2025, which described persistent disease only at the right femoral area, marked with a blue arrow. Vertebral lesions are marked with a V, sternal lesions with an S, and the femoral metastasis with an F. The sacral soft tissue mass is limited by a square.

There was a size reduction of the vulvar lesion after two cycles of treatment with ribociclib (now 2.5 x 1 cm) and a significant decrease of the CA 15-3 tumor marker (Figure 4). The first assessment of therapy response was conducted in November 2024, with a PET scan that revealed a reduction in the extent and intensity of contrast uptake in the pre-existing bone lesions. After seven cycles of treatment, a new PET scan, performed in April 2024, only identified the persistence of a bone metastasis of lytic nature in the trochanteric region of the right femur, with a reduction in the degree of metabolic activity compared to the previous study, thus confirming a good response to treatment, which the patient is maintaining at the time of writing (Figure 5).

## Discussion

The carcinoma of the vulva accounts for 4% of gynecological neoplasms. Of these, adenocarcinomas occur in 0.1% of the cases and, within this group, mammary gland-like adenocarcinomas are very rare [1]. As for their origin, there are two theories described in the literature: origin in tissue remaining from the embryonic mammary ridge, or origin in glands attached to the skin in the anogenital region, with a structure similar to the mammary gland. The first case of vulvar mammary-like adenocarcinoma was reported by Greene in 1936. Since then, around an additional 45 cases have been described in the literature [1,2,5-8].

When it comes to age at diagnosis, the cases reported occurred in women aged between 45 and 90 years old. Clinically, in most cases, this neoplasm initially manifests as an asymptomatic nodule, taking months to years to grow and consequently generate symptoms [1].

Irvin et al. defined the criteria for diagnosing this disease [2]. When an in situ component is not present, and in the absence of concurrent breast carcinoma, the following should be sufficient to categorize the lesion of primary vulvar origin: (1) a morphologic pattern consistent with breast carcinoma; (2) the presence of estrogen and progesterone receptors; and/or (3) positivity for common breast cancer markers such as epithelial membrane antigen, carcinoembryonic an­tigen, and glandular keratins. Based on these criteria, we diagnosed this disease as an adenocarcinoma of mammary-like glands in the vulva. Besides these criteria, primary vulvar adenocarcinoma can be distinguished from metastatic breast cancer based on the presence of residual normal mammary glands adjacent to the primary tumor, while no normal mammary tissue is observed in metastatic cancer [5].

As for the extent of the disease at diagnosis, most of the reported cases refer to localized tumors, with surgical treatment and the possibility of adjuvant systemic treatment afterwards. These carcinomas may behave more aggressively than their counterparts in the breast, with an estimated 60% metastatic rate to regional lymph nodes. Rare cases of distant metastasis have been reported, such as the present case. In this case, we have a mammary-like adenocarcinoma of the vulva with diffuse bone metastasis at diagnosis [1].

Given the rarity of this type of tumor, there are no guidelines for its treatment, so it was extrapolated from the guidelines for the treatment of hormone receptor-positive and HER-2-negative metastatic breast cancer. For these patients, the MONALEESA-2 trial showed an improvement in overall survival (OS) and progression-free survival (PFS). Adding ribociclib to an aromatase inhibitor resulted in a median OS of 63.9 months (95% confidence interval (CI): 52.5-71), with around a 12-month benefit compared to letrozole and placebo (hazard ratio (HR) 0.76, 95% CI: 0.63-0.93). The median PFS was 25.3 months with ribociclib, with a benefit of nine months compared to placebo (HR 0.56, 95% CI: 0.43-0.72) [9]. In this patient's case, there was an excellent response in the PET scan, with a reduction of the extent of bone lesions - after seven cycles of treatment, she only has disease at the femoral level, and maintains response until the time of writing the present article.

## Conclusions

This is a rare histological type of vulvar carcinoma, presenting atypically with synchronous diffuse bone metastatic dissemination. The decision for systemic treatment was guided by the histology, and there was a clear and sustained metabolic response to therapy. Documenting this case is essential to provide the medical community with knowledge for the management of similar cases in the future.
